# Re-induction of the cell cycle in the *Arabidopsis* post-embryonic root meristem is ABA-insensitive, GA-dependent and repressed by *KRP6*

**DOI:** 10.1038/srep23586

**Published:** 2016-03-29

**Authors:** Jeroen Nieuwland, Petra Stamm, Bo Wen, Ricardo S. Randall, James A. H. Murray, George W. Bassel

**Affiliations:** 1 School of Biosciences, Sir Martin Evans Building, Cardiff University, Museum Avenue, Cardiff CF10 3AX, Wales, UK; 2 School of Applied Sciences, University of South Wales, Pontypridd, CF37 4AT, United Kingdom; 3 School of Biosciences, University of Birmingham, Birmingham B15 2TT, United Kingdom

## Abstract

Seeding establishment following seed germination requires activation of the root meristem for primary root growth. We investigated the hormonal and genetic regulation of root meristem activation during *Arabidopsis* seed germination. In optimal conditions, radicle cell divisions occur only after the completion of germination and require *de novo* GA synthesis. When the completion of germination is blocked by ABA, radicle elongation and cell divisions occurred in these non-germinating seeds. Conversely under GA-limiting conditions, ABA-insensitive mutants complete germination in the absence of radicle meristem activation and growth. Radicle meristem activation and extension can therefore occur independently of completion of the developmental transition of germination. The cell cycle regulator *KRP6* partially represses GA-dependent activation of the cell cycle. Germination of *krp6* mutant seeds occurs more rapidly, is slightly insensitive to ABA in dose-response assays, but also hypersensitive to the GA synthesis inhibitor PAC. These conflicting phenotypes suggest the cell cycle uncouples GA and ABA responses in germinating *Arabidopsis* seeds, and that *KRP6* acts downstream of GA to inhibit mitotic cell cycle activation during germination.

Within the seed of most plant species is a complete mature embryo^1^. Following the breaking of dormancy, the process of germination is initiated. Germination of *Arabidopsis* seeds is a two-step process^2^,^3^, the first being manifest as the rupturing of the surrounding seed coat (testa), and the second being the protrusion of the radicle through the endosperm marking the completion of germination. This also encompasses the transition of the embryo into a seedling^4^. Growth from embryo to seedling is primarily driven by cell expansion events in the embryonic axis (radicle and hypocotyl)^5^. Following germination, most future growth of the plant is dependent on cell divisions occurring in both the root and shoot meristems contained within the mature plant embryo.

Activation of the embryo root meristem is necessary for the initiation of root growth and development, and is a key component of seedling establishment. Rapid meristem activation defines both seedling survival and seedling vigour in an agricultural context^6^. A rapidly downward growing root assists the establishing seedling in obtaining a reliable water supply and avoiding drought stress as the water line drops following a germination-stimulating rainfall in the field^6^. This process is therefore central to crop establishment and food security under variable environmental conditions.

Despite the central role post-embryonic meristem activation plays in plant agriculture, there is surprisingly little known about how this process is regulated. Many factors have been described in cellular patterning during root development^7^, but less is known as to how these are regulated in a germinating embryo, or how their induction is controlled.

The activation of the mitotic cell cycle has been previously demonstrated to occur in the shoot and root meristems during the final stages of *Arabidopsis* seed germination^8^,^9^ and to be dependent upon the hormone gibberellic acid (GA)^10^,^11^. The transcription factors *TCP14* and *TCP15* have been demonstrated to promote GA-mediated cell divisions in the germinating radicle^9^. A role for these cell divisions in the promotion of axis elongation driving the protrusion of the radicle through the seed coat, marking the completion of germination *sensu stricto*, has been proposed^8^. Less clear is the role of the endocycle in germination, although the presence of endocycles has been reported to occur early in the germination process^8^. This is the process of nuclear DNA replication leading to genome doubling without an intervening mitosis, and is frequently associated with expansion driven growth. *KIP RELATED PROTEIN5* (*KRP5*), which encodes a cyclin-dependent kinase inhibitor, has been reported to limit the rate at which radicle emergence occurs during *Arabidopsis* seed germination^12^ through the control of endoreduplication, although the relationship between *KRP5* and post-embryonic radicle meristem activation was not established.

To better understand the control of root meristem activation during the seed to seedling transition, we explored the hormonal and genetic regulation of post-embryonic induction of the cell cycle in germinating *Arabidopsis* seeds.

## Results

### Hormonal control of embryonic root meristem activation

**Figure 1:**
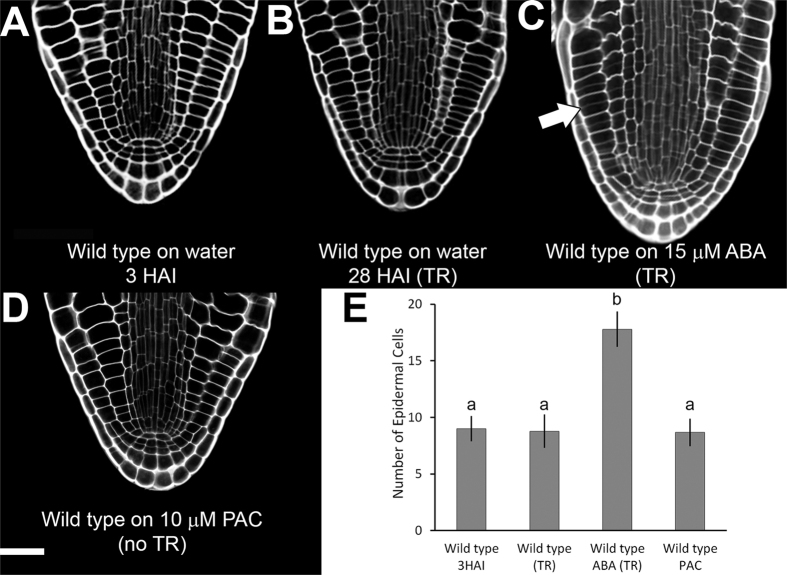
Hormonal regulation of cell cycle progression in the embryo to seedling transition. (**A**) Cellular anatomy of the radicle in a mature *Arabidopsis* embryo at 3 HAI. (**B**) *Arabidopsis* embryo from a seed at 28 HAI, immediately after testa rupture, and before the completion of seed germination. (**C**) *Arabidopsis* embryo from a seed imbibed on 15 μm ABA for 7 days. New cell walls can be observed in the radicle as having lighter staining, indicated by the white arrow. (**D**) *Arabidopsis* embryo from a seed treated with 10 μm PAC for 7 days. (**E**) Number of epidermal cells for the embryos shown in (**A–D**), n = 9 and error bars are SD. White scale bar in (**A**) is 50 μm. Same letters above bars indicate no significant difference, while different letters suggest a significant difference (p < 0.01).

The activation of the embryonic root meristem during germination was anatomically investigated using the previously described mPA-PI staining method to visualize the creation of new plant cell walls by confocal microscopy^13^,^14^. The radicle, a sub-region of the embryonic axis covered by a surrounding root cap has roughly 8 epidermal and cortical cells (Fig. 1A). When imbibed on water at 22 °C, by 28 hours after imbibition (HAI) seeds reach a stage immediately prior to completion of germination, with the seed coat (testa) ruptured, but the endosperm still intact. The cellular anatomy of the radicle remains visibly unchanged at this stage (Fig. 1B). Thus, under optimal conditions, the completion of cell division resulting in new cell plate formation does not occur in seeds before the completion of germination.

**Figure 2:**
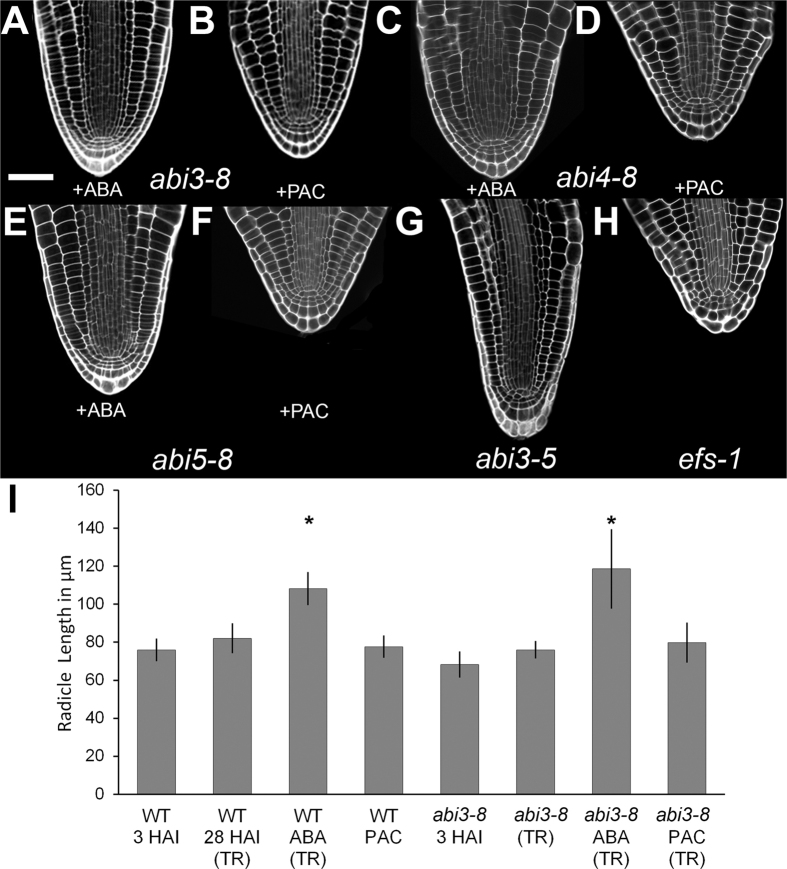
Genetic and hormonal control of radicle elongation. Cellular anatomy of the *abi3-8* mutant on (**A**) 15 μm ABA and (**B**) 10 μm PAC. *abi4-8* mutant on (**C**) 15 μm ABA and (**D**) 10 μm PAC. *abi5-8* mutant on (**E**) 15 μm ABA and (**F**) 10 μm PAC. All treatments in samples (**A**–**F**) were for 7 days and seeds which had testa ruptured were selected. (**G**) Anatomy of viviparous *abi3-5* embryos at 3 HAI. (**H**) Anatomy of viviparous *efs-1* embryos at 3 HAI. (**I**) Radicle lengths of embryos in (**A–F**) and Fig. 1(A–D), n = 9 and error bars are SD. White scale bar for all in (**A**) is 50 μm. Asterisks indicate significant differences (two-tailed Student’s t-test, p < 0.05). P-values for all pairwise comparisons are in Supplementary Table 2.

The germination of *Arabidopsis* seeds can be blocked by imbibing them in 15 μM ABA^15^. Following 7 days of incubation on this inhibitory hormone, step 1 of germination was completed, with the testa ruptured^16^, but endosperm rupture and thus completion of germination was inhibited. In these seeds, cells in the root cap, epidermis, cortex and endodermis are dividing, as indicated by the presence of new cell plates (Fig. 1C). Concurrent with these cell divisions the elongation of the radicle is observed (Fig. 2I), and a doubling of epidermal cell number (Fig. 1E). ABA therefore blocks step 2 and hence the completion of germination, but does not inhibit the activation of the root meristem as part of seedling establishment. These observations demonstrate that these processes are separable, and both cell divisions and elongation of the radicle can occur independently of the completion of *Arabidopsis* seed germination^17^.

Testa rupture of *Arabidopsis* seeds does not occur when they are imbibed in the GA-synthesis inhibitor paclobutrazol (PAC)^16^,^18^, demonstrating that *de novo* GA synthesis is required to initiate cell expansion and step 1 of germination in seeds. Cell divisions are not visible in PAC-imbibed seeds after 7 days (Fig. 1D) and the radicle does not elongate under these conditions (Fig. 1E). These data are consistent with the requirement for GA to both promote germination, and to induce divisions in the radicle during germination^9^. In contrast, cell divisions in the root meristem becomes apparent when the completion of germination is blocked by ABA. Here, the GA stimulus to induce the germination process and differentiation into a seedling^19^, including the activation of the cell cycle in the root meristem, is present. However the protrusion of the radicle through the endosperm is limited by the activity of ABA^20^.

### Elongation of the radicle and cell divisions require GA and are promoted by ABA signalling factors

Inhibition of germination by ABA did not prevent activation of cell division within the radicle during wild type seed germination. We explored the role of downstream ABA signalling in the control of post-embryonic meristem activation by examining mutant seeds which are insensitive to this hormone^21^.

Activation of cell divisions within the radicle of *ABA-INSENSITIVE3* (*ABI3*), *ABI4* and *ABI5* mutant seeds^21^ was examined. These three signaling components are transcription factors downstream of ABA perception, and their absence renders seeds less sensitive to the inhibitory effects of ABA on germination. Treatment of mutant seeds with 15 μM ABA did not inhibit the completion of germination, as occurred with wild-type seeds, but did delay radicle protrusion. After 7 days of imbibition on ABA, when seeds were testa ruptured, prior to completion of germination, radicle cell divisions were observed in each of the *abi3-8, abi4-8* and *abi5-8* mutant embryos (Fig. 2A,C,E). Here cells divided in half, representing one round of cell divisions resulting in a doubling of cell number as also occurred in the wild type imbibed in ABA for 7 d (Fig. 1C,E). In the *abi3-8* mutant^22^, these cell divisions coincided with elongation of the radicle which extended to a greater degree than equivalent wild-type seeds (Fig. 2I, significant difference according to a Student’s t-test, p < 0.05). The treatment of *abi* mutants with ABA therefore resulted in enhanced radicle elongation, but did not affect the cell divisions which occurred prior to the completion of germination in both WT and *abi* backgrounds.

Cell divisions in all *abi* mutants are limited to cells underlying the root cap and do not extend beyond the domain of the radicle. ABA insensitivity conferred by *abi3-8, abi4-8* or *abi5-8* therefore does not alter the domain of cell divisions in the radicle meristem.

In addition to being less sensitive to ABA, *abi* mutant seeds are able to germinate on concentrations of the GA synthesis inhibitor paclobutrazol (PAC) that inhibit the wild type^16^. The induction of cell expansion and thus germination in these *abi* mutants therefore has a reduced requirement for *de novo* GA synthesis^21^.

Imbibing wild-type seeds on 10 μM PAC blocked both testa rupture and germination, as well as radicle cell division and elongation (Fig. 1). Testa rupture and germination were not blocked by 10 μM PAC in *abi3-8, abi4-8* or *abi5-8* mutants, but these events were strongly delayed. Imaging PAC-imbibed *abi3-8* mutant seeds that showed testa rupture at 7 d revealed that radicles did not elongate, and cells in the radicle did not divide (Fig. 2B). This is in contrast to *abi3-8* mutant seeds imbibed on 15 μM ABA which did not inhibit their germination, and both cell division and radicle elongation were observed (Fig. 2A).

Similar observations were made with *abi4-8* and *abi5-8* mutants imbibed on 15 μM ABA and 10 μM PAC. In all cases, cells in the radicle had divided just prior to completion of germination in ABA-treated seeds (Fig. 2C,E), while embryos completed germination in the absence of the activation of cell divisions in PAC-imbibed seeds (Fig. 2D,F).

These observations demonstrate that each of the *abi3-8, abi4-8* and *abi5-8* mutations reduce the sensitivity of germination to the inhibitory effect of ABA, but do not remove the requirement of GA for the activation of mitotic cell divisions in the root meristem. Therefore, radicle elongation and cell divisions in the radicle are not required for germination, and conversely the completion of germination is not required for meristem activation. Germination may therefore be completed with no radicle extension. These two processes are likely independent of each other, and possess different hormonal sensitivities.

### Vivipary in the *abi3-5* mutant is associated with premature activation of the radicle meristem

The *abi3-5* mutant is a strong *abi3* null allele, having green seeds and limited desiccation tolerance^23^. These mutant seeds germinate prior to completion of development (vivipary). We examined the cellular anatomy of viviparous *abi3-5* embryo radicles, and found that they had undergone extensive cell divisions and elongation (Fig. 2G). This demonstrates that the premature embryo to seedling transition occurring in this highly ABA-insensitive mutant is accompanied by ectopic activation of the radicle meristem during embryo development. *ABI3* therefore acts during seed development to limit a developmental programme that includes root meristem activation as part of its suppression of seedling traits^24^.

The *EARLY FLOWERING IN SHORT DAYS1* (*EFS1*) *efs-1* mutant was previously reported to show vivipary^25^. The cellular anatomy of this radicle was also examined. In this mutant, cell divisions indicative of premature meristem activation were not observed. Premature activation of the root meristem is therefore not necessarily associated with precocious germination. This is consistent with our earlier conclusion that meristem activation is independent of the completion of germination.

### *KRP6* transcription declines during germination and alters GA sensitivity

**Figure 3:**
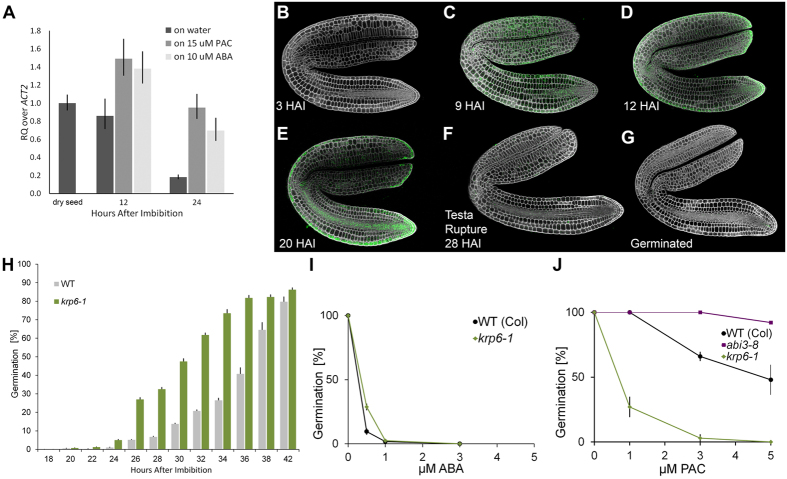
*KRP6* gene and protein dynamics, and mutant germination phenotypes. (**A**) *KRP6* expression pattern during *Arabidopsis* seed germination. Expression levels were determined by qRT-PCR with RNA obtained from dry seeds, and after 12 and 24 h of imbibition on water, 15 μM ABA or 10 μM PAC. n = 3 and error bars are SD. (**B–G**) Spatiotemporal pattern of KRP6 protein abundance visualized using a *KRP6::KRP6-GUS* translational fusion at (**B**) 3 HAI, (**C**) 9 HAI, (**D**) 12 HAI, (**E**) 20 HAI, (**F**) testa rupture (28 HAI) and (**G**) a recently germinated embryo. (**H**) Rate of *krp6-1* seed germination relative to a wild-type Ws control under optimal conditions at 22 °C. n = 5 and error bars are SEM. (**I**) ABA dose-response of *krp6-1* mutant seeds. (**J**) PAC dose-response of *krp6-1* mutant seeds. n = 4 and error bars are SD.

Gene expression associated with cell cycle control in *Arabidopsis* has been previously investigated over a time-course of seed germination^8^, but not all cell cycle genes are represented by the ATH1 Affymetrix microarray chip. One such gene encodes the cell cycle inhibitor KIP-RELATED PROTEIN 6 (KRP6). Using qRT-PCR we examined the expression profile of *KRP6,* which was found to decline during *Arabidopsis* seed germination (Fig. 3A). The precipitous decline of this transcript suggested a potential role for *KRP6* in cell cycle control during the seed to seedling transition. In seeds imbibed on 15 μM ABA, *KRP6* expression levels are higher than in water-imbibed seeds, and remain high at 24 HAI. A similar expression pattern is observed in seeds treated with 10 μM PAC. This suggests that *KRP6* is down regulated on the completion of step 2 of germination, which does not happen in either of these conditions.

### KRP6 expression pattern and protein dynamics

We examined the spatial and temporal regulation of the KRP6 protein using a C-terminal *KRP6::KRP6-GUS* translational fusion reporter. In the early stages of seed germination, the protein was not detectable up to 3 HAI (Fig. 3B). By 9 HAI the protein showed a broad distribution across the embryo with the exception of the radicle where it was absent (Fig. 3C). By 12 HAI the protein had spread to the radicle (Fig. 3D) where it persisted until 20 HAI (Fig. 3E). Upon the onset of testa rupture at 28 HAI, the protein had largely disappeared from the embryo (Fig. 3F) and remained undetectable in embryos that had recently completed germination (Fig. 3G). The initial induction pattern of the KRP6 protein does not match the RNA abundance profile (Fig. 3A), suggesting additional post transcriptional regulation, although the decline in protein abundance by testa rupture is consistent.

### Functional role of *KRP6* in seed germination

To examine the functional role of *KRP6* in the control of germination, we obtained a T-DNA mutant, termed *krp6-1*, where expression of this gene was strongly reduced (Supplementary Figs 1 and 2). Germination of this mutant was faster than that of wild-type under optimal conditions (Fig. 3H). In an ABA dose-response germination assay, the *krp6-1* was slightly insensitive to ABA (Fig. 3I). Conversely, *krp6-1* mutant seeds were strongly hypersensitive to PAC (Fig. 3J), suggesting an increased requirement for GA-synthesis for their germination.

### *KRP6* represses the GA-mediated induction of the cell cycle during Arabidopsis seed germination

To investigate the role of *KRP6* on the induction of the cell cycle during *Arabidopsis* seed germination, the activation of the cell cycle across this developmental transition was measured by scoring mitotic figures as previously described^8^.

**Figure 4:**
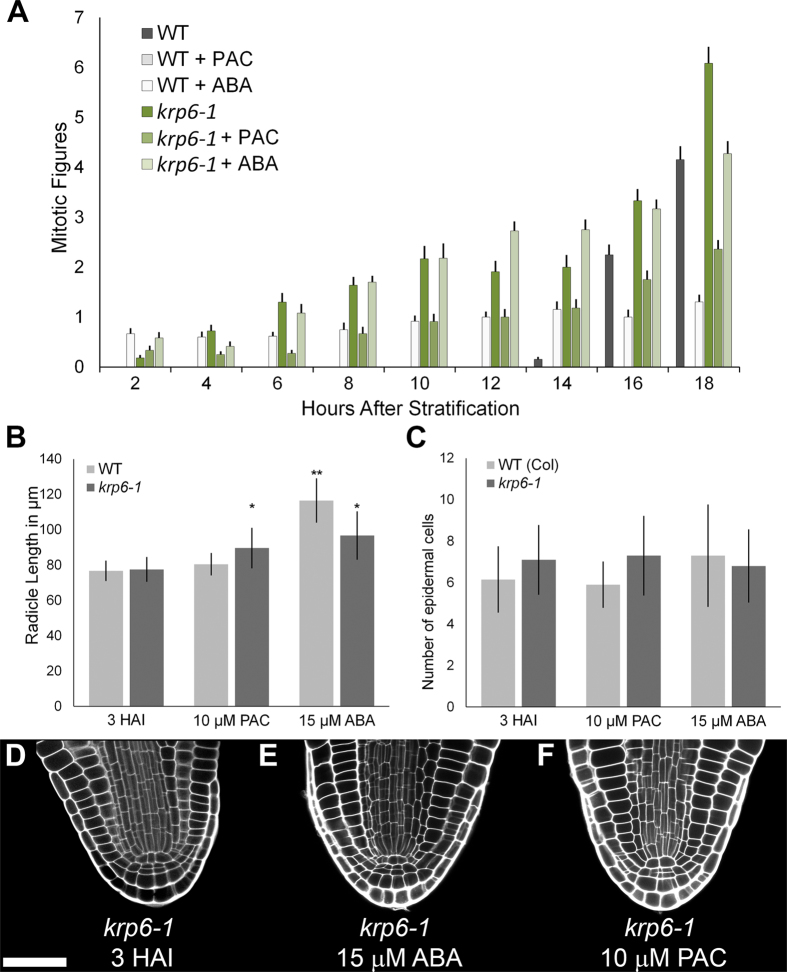
Regulation of mitotic index and radicle meristem activity by *KRP6*. (**A**) Mitotic figures of wild type (*Col*) and *krp6-1* mutant seeds over a time course of seed germination imbibed in water, 15 μm ABA or 10 μm PAC. n = 12 and error bars are SD. (**B**) Radicle length in wild type and *krp6-1* mutant embryos treated with 15 μm ABA and 10 μm PAC for 7 days. (**C**) Same as (**B**) counting the number of epidermal cells. For (**B**,**C**) n = 10 and error bars are SD. Asterisks indicate significant differences when compared to corresponding embryos at 3 HAI, with one asterisk indicating p < 0.05, and two asterisks p < 0.01. For a full table of p-values for all pairwise comparisons, see Supplemental Table 3. (**D**) *krp6-1* mutant radicle at 3 HAI. (**E**) *krp6-1* mutant embryo treated with 15 μm ABA for 7 days and (**E**) 10 μm PAC for 7 days. White scale bar in (**D**) is 25 μm.

Wild type seeds show a progressive increase in mitotic figures from 14 HAI onwards, while imbibing these seeds in ABA stimulated premature induction of the mitotic cell cycle from the earliest time point measured (2 HAI) onwards (Fig. 4A). Treatment of wild type seeds with PAC abolished the onset of the mitotic cell cycle during germination, demonstrating the requirement of GA for the induction of this process^9^,^10^.

The *krp6-1* mutant showed ectopic induction of mitotic figures at early stages of seed germination relative to the wild type control on water, and also showed a higher mitotic index at later stages of germination (Fig. 4A). Unlike wild-type seeds, treatment of *krp6-1* seeds with a concentration of PAC sufficient to block germination reduced but did not prevent induction of the cell cycle. The mitotic index of PAC-treated embryos was not as high as the water-imbibed mutant seed control, but was not reduced to the same extent as in the wild type. Taken together, these results demonstrate KRP6 to be a repressor of GA-mediated induction of the mitotic cell cycle during *Arabidopsis* seed germination.

### Control of radicle elongation and root meristem cell division by *KRP6*

The role of *KRP6* in the control of cell division and radicle elongation was determined by microscopically examining radicle growth in the mutant in response to ABA and PAC treatment. In the wild type, radicle length significantly increased upon ABA-treatment, but not on PAC treatment, whereas radicle length in *krp6* mutant embryos increased significantly when treated with either hormone (Fig. 4B). The number of epidermal cells in the radicle was not significantly affected in wild type or the *krp6* mutant by either treatment (Fig. 4D–F). These data suggest that the *krp6* mutation leads to an increase in mitotic figures in untreated embryos, and in those treated with ABA or PAC along with a slight but significant increase in radicle length. This raises the possibility that cells in the radicle of the *krp6* mutant are stuck in mitosis possibly due to a requirement for GA to complete cytokinesis.

## Discussion

Understanding the mechanisms which drive the seed to seedling transition is of central importance to enhancing food security during a period of rapid climate change^26^. Seedling establishment is a particularly vulnerable period of crop production, and the absence of plants in the field eliminates the possibility of future harvest. Rainfall in the field promotes the germination of seeds, after which point the water level in the soil begins to travel further down the soil column. Rapid activation of the root meristem and downward growth of this organ can enhance seedling survival by ensuring a water supply for the plant during this potentially water limiting stage of plant growth.

Previous reports have characterized the induction of both the mitotic cell cycle and the endocycle in the radicle meristem during *Arabidopsis* seed germination^8^ and its dependence on GA and TCP transcription factors^9^.

Under optimal laboratory conditions, these cell divisions in the radicle are not detected until after the completion of germination (Fig. 1B). Inhibition of seed germination by application of ABA, a hormone produced in response to stress conditions, seems to specifically inhibit the second and final step of germination, marked by protrusion of the radicle through the endosperm^20^. However, ABA treatment did not prevent cell divisions in the radicle meristem (Fig. 1C). We suggest that treatment of seeds with ABA blocks the completion of germination (radicle protrusion), but not the progression of the seedling developmental program within the embryo. This has been previously reported for the *abi4* mutant treated with ABA, where cotyledons turned green under these conditions^19^.

The maintenance of embryonic traits by ABA signalling, specifically involving *ABI3*, has been reported^27^. Premature activation of the radicle meristem was seen in the strong *abi3-5* mutant in viviparous embryos, and has been reported for the shoot meristem in another strong *abi3* mutant^24^. These findings demonstrate that ABA appears to suppress the embryo to seedling transition via *ABI3*, by suppressing meristem activation associated with seedling traits.

Cell divisions in the radicle were not prevented by application of ABA to wild-type seeds (Fig. 1C) and ABA treatment led to the premature appearance of mitotic figures (Fig. 4A). However, the treatment of seeds with PAC did block the cell cycle. Collectively, these data confirm that the induction of division in the radicle meristem during germination requires GA, and is not blocked by ABA. This is in contrast to the effect of ABA on cultured tobacco cells, where this hormone treatment blocked entry into the cell cycle^28^.

The effect of ABA on radicle meristem activation was examined using *abi* mutants. Germination of these *abi* mutants was shown to be insensitive to exogenous ABA, and requires less *de novo* GA synthesis^29^. However, *abi* mutations did not reduce the GA requirement for the induction of the mitotic cell cycle in the radicle meristem (Fig. 2B,D,F). These mutations did however reduce the need for *de novo* GA synthesis for cell expansion, as has been reported previously^29^. Together these data demonstrate that meristem activation and radicle elongation are not required for seed germination, but also that completion of germination is not required for meristem activation. This is consistent with a previous report where it was shown that the *shortroot* mutant, in which cell divisions are not induced in the radicle, does not exhibit any germination defects^30^.

The growth of the radicle in ABA-treated seeds which do not complete germination also demonstrates that radicle elongation can occur independently of the completion of germination. Expansion of the cortical cells of the radicle during germination has been previously quantified^5^ while elongation of live-imaged root cap cells was not detected^17^. The elimination of radicle-based growth in *abi* mutants treated with PAC suggests that these seeds complete germination by increased elongation of the hypocotyl. The growth required to drive germination is most likely compensated by hypocotyl growth in the absence of that contributed by the radicle. Multiple adaptive spatiotemporal patterns of cell expansion may enable the completion of germination in *Arabidopsis* embryos, suggesting this transition is not deterministic.

The *KRP6* gene represses GA-mediated induction of the mitotic cell cycle in germinating *Arabidopsis* embryos (Fig. 4A). Although embryos of the *krp6-1* mutant show increased mitotic figures within the radicle during ABA treatment, no significant increase in cell number was observed (Fig. 4C,E). This suggests that mitosis is not being completed in these conditions, perhaps due to a further GA-dependent or KRP6-dependent event in these early mitoses. Hence KRP6 acts to inhibit the mitotic cell cycle, but the pthe premature entry into the mitotic cycle that its loss provokes can lead to a subsequent delay during the process of mitosis itself. A previous report suggested a relationship between KRP6 expression and mitotic progression, showing that overexpression of KRP6 could block mitotic completion and cytokinesis when overexpressed in the giant cells produced on root knot nematode infection. Whilst it is difficult to draw conclusions from the phenotype caused in these specialised cells compared to the germinating embryo, these results demonstrate an interaction between mitotic processes and KRP6, albeit from opposite changes in KRP6 expression^31^.

Hypersensitivity of the germination of *krp6-1* seeds to PAC treatment demonstrates a positive role for this gene in the promotion of GA-stimulated germination (Fig. 3J), though this does not appear to occur through the premature completion of cell divisions in the radicle (Fig. 4B–E). These observations suggest that *KRP6* is likely playing different roles in the control of germination and the activation of the embryonic root meristem. This could be consistent with KRP6 having a role in controlling endocycles associated with expansion growth during germination, as well as in the subsequent activation of cell division.

Previous work on both *KRP6* and other *KRP* genes in Arabidopsis has shown that their overexpression in plants leads to decreased cell division leading to serrated leaves composed of larger cells that show a higher degree of endocycling, due to the inhibition of the mitotic cycle by KRPs^32^,^33^. Moreover the effect of KRPs are dependent on their expression level: at high level they inhibit both mitotic and endocycles, whereas at modest levels of expression they inhibit selectively mitotic cycles^34^. This differential effect of KRPs at different expression levels may explain the different phenotypes of *krp6*.

We therefore propose the following model for KRP6 function during germination. Initially, as we have observed KRP6 is present at high levels, blocking both the endocycles associated with cell growth and mitotic division. As germination initiates, KRP6 levels fall, allowing endocycles to occur associated with the cell growth driving germination. Late in germination KRP6 levels becomes almost undetectable, allowing mitotic cycles to initiate. Hence KRP6 could therefore normally play a role in inhibiting the premature onset of mitotic cycles in the radicle meristem, whilst promoting the GA-dependent cell expansion perhaps through endocycling of the cortical cells behind the root cap. In this model, the faster germination of *krp6* mutants therefore results from earlier activation of endocycling, and incidentally an earlier activation of mitotic cycling.

The hypersensitivity to PAC of *krp6* mutants suggests that their early activation of growth is dependent on GA. In this regard it is interesting to note that KRP6 is also involved in integrating energy homeostasis and cell cycle control through the SNF1-related protein kinase–1 SnRK1^33^, suggesting complex potential interactions between hormonal regulation, energy status sensing and KRP6 levels during coordination of the germination process.

Further work linking the control of *KRP6* to previously described root development mechanisms will be valuable in understanding how this process is regulated as well as understanding the mechanistic relationship between the control of the cell cycle and completion of germination.

## Methods

### Seed materials

All genotypes used were in a Columbia background with the exception of the *efs-1* mutant which is Landsberg *erecta. krp6-1* mutant seeds were obtained from CropDesign, Gent.

Plants were grown with 16 h light (light intensity 150–175 μmol⋅m^−2^⋅s^−1^) at 23 °C and 8 h dark at 18 °C. Plants were harvested when flowering ceased and were stored in glassine bags for 1 month at 24 °C to remove primary dormancy. Seeds were cleaned through a 500 μm mesh, and used for further experiments.

### Germination assays

Germination assays were performed as previously described by surface-sterilizing seeds in 10% parazone and placing them onto plates of ½ MS media containing 0.8% (w/v) PGP agarose^25^. Each assay was repeated three times with at least 100 seeds. Germination was scored 7 days after imbibition.

### Hormone treatment

*Arabidopsis* seeds were surface-sterilised with 10% parazone, and placed onto plates of ½ MS medium with 0.8% agar (w/v), supplemented with hormones as indicated.

For the imaging of radicles, media was supplemented with either 15 μM abscisic acid (ABA; Sigma), or 10 μM paclobutrazol (PAC; Fluka). After 7 days of incubation, embryos from *Arabidopsis* seeds were dissected with a scalpel and forceps under a binocular microscope.

### Confocal microscopy and image analysis

Dissected embryos were stained and cleared as previously described using the mPS-PI technique^5^,^14^, and imaged using a Zeiss LSM 710 confocal microscope with the Zeiss Zen software. Radicle lengths and cell number were determined on 2D images of embryo radicles using ImageJ software. For each treatment, 10 embryos were analysed. The number of epidermal cells underlying the root cap was counted. Radicle length was determined as the distance from the quiescent centre to the first cells of the inner cortex.

### Mitotic index analysis

Mitotic index analysis was performed as previously described^8^. Germinating embryos were fixed overnight in FAA (3.7% paraformaldehyde/81% EtOH/5% glacial acetic acid), rinsed with water, and mounted under cover slips. After crushing, the samples were snap-frozen with liquid nitrogen to allow the removal of the coverslip and mounted in Vectashield with DAPI (Vector Laboratories, Burlingame, CA). The samples were examined with a Zeiss Axiophot fluorescent microscope, and the number of metaphases and anaphases were scored for each embryo. At least 12 embryos were counted for each sample.

### Cloning of GUS reporter construct

A genomic fragment of the *KRP6* gene was amplified using PCR with the primers: fwd- 5′-GGGGACAAGTTTGTACAAAAAAGCAGGCTTCATATATTATCTATTTAAAC-3′ and rev-5′-GGGGACCACTTTGTACAAGAAAGCTGGGTCAAGTCGATCCCACTTGTAGCG-3′ as forward and reverse primers respectively. The fragment was cloned using Gateway Technology (Invitrogen) into the pMDC162 vector containing the GUS coding sequence^35^.

### Imaging of GUS reporters

Staining for GUS activity was performed in staining buffer (sodium phosphate buffer pH 7.0, 2 mM 5-bromo-4-chloro-3-indolyl-β-D-glucuronic acid (Sigma), 1 mM potassium ferro- and ferricyanate) at 37 °C until a blue product was visible. GUS-stained embryos were subsequently fixed, cleared and mPA-PI stained as described earlier^5^,^14^ for confocal imaging. GUS crystals were imaged using “reflectance” settings on a second channel using a Zeiss LSM 710 microscope.

### qRT-PCR of *KRP6*

qRT-PCR was performed as described previously^8^ using CAAGCACAAGCTTCTCACCA and GTGAAACAACCGGAGCTGAT as forward and reverse primers, respectively. *ACTIN2* was used a reference transcript, amplified with ACATTGTGCTCAGTGGTGGA and CTGAGGGAAGCAAGAATGGA primers. RNA was isolated from dry seeds as control, and from seeds imbibed on water, 10 μM PAC, and 15 μM ABA after 12 and 24 hours each, and transcribed into cDNA with the “GoScript Reverse Transcription System” (Promega). qRT-PCR was performed using the “PerfeCTa^®^ SYBR^®^ Green FastMix^®^” (Quanta BioSciences Inc.). Data were derived from three biological replicates.

## Additional Information

**How to cite this article**: Nieuwland, J. *et al*. Re-induction of the cell cycle in the *Arabidopsis* post-embryonic root meristem is ABA-insensitive, GA-dependent and repressed by *KRP6. Sci. Rep.* **6**, 23586; doi: 10.1038/srep23586 (2016).

## Supplementary Material

Supplementary Information
