# Supplementary material for: Re-induction of the cell cycle in the Arabidopsis post-embryonic root meristem is ABA-insensitive, GA-dependent and repressed by KRP6
Source: Sci Rep. 2016 Mar 29;6:23586. doi: 10.1038/srep23586 (PMC4810365; doi:10.1038/srep23586)
Supplement: Supplementary Information [file srep23586-s1.docx]

**Supplementary Information**

**Re-induction of the cell cycle in the *Arabidopsis* post-embryonic root meristem is ABA-insensitive, GA-dependent and repressed by *KRP6***

Jeroen Nieuwland^1,2,+^, Petra Stamm^3,+^, Bo Wen^1,§^, Ricardo S. Randall^1^, James A.H. Murray^1^, George W. Bassel^3,*^

***krp6-1***

**Supplementary Figure 1.** Schematic of the T-DNA insertion in the *krp6-1* allele. The T-DNA insertion is present in the first exon (black box) of the open reading frame indicated by the black triangle.


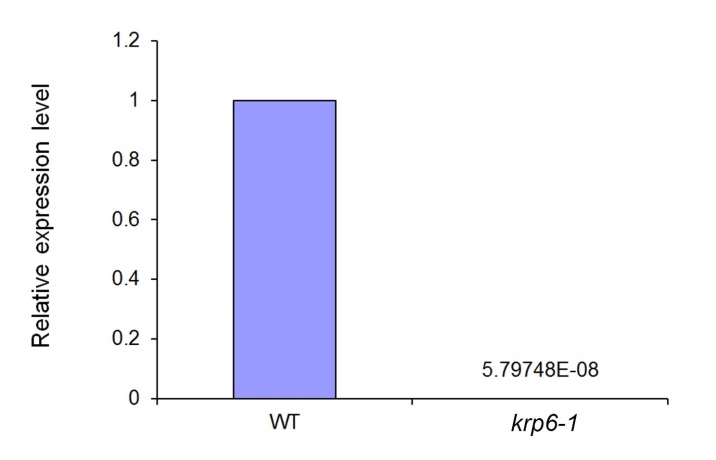


**Supplementary Figure 2.** qRT-PCR confirming a decrease in *KRP6* transcripts in 4-day-old seedlings for each the wild type and *krp6-1* mutant.

**Supplementary Table 1.** P-values for pairwise comparisons of epidermal cell numbers in WT radicles as shown in Figure 1E. A two-tailed Student’s t-test was performed for each pair, and the respective p-value is given.

|  | **WT 3HAI** | **WT TR** | **WT ABA TR** | **WT PAC** |
| --- | --- | --- | --- | --- |
| **WT 3HAI** | N.A. | 7.2·10^-01^ | 3.0·10^-10^ | 5.5·10^-01^ |
| **WT TR** | 7.2·10^-01^ | N.A. | 1.1·10^-09^ | 8.6·10^-01^ |
| **WT ABA TR** | 3.0·10^-10^ | 1.1·10^-09^ | N.A. | 2.8·10^-10^ |
| **WT PAC** | 5.5·10^-01^ | 8.6·10^-01^ | 2.8·10^-10^ | N.A. |

**Supplementary Table 2.** P-values for pairwise comparisons of radicle lengths as shown in Figure 2I. A two-tailed Student’s t-test was performed for each pair, and the respective p-value is given.

|  | **WT 3 HAI** | **WR TR (28 HAI)** | **WT ABA TR (7d)** | **WT PAC (7d)** | ***abi3-8* 3 HAI** | ***abi3-8* TR** | **abi3-8 ABA TR (7 d)** | **abi3-8 PAC TR (7 d)** |
| --- | --- | --- | --- | --- | --- | --- | --- | --- |
| **WT 3 HAI** | N.A. | 7.8·10^-02^ | 2.3·10^-08^ | 6.4·10^-01^ | 2.1·10^-02^ | 9.9·10^-01^ | 1.4·10^-05^ | 3.8·10^-01^ |
| **WR TR (28 HAI)** | 7.8·10^-02^ | N.A. | 1.8·10^-06^ | 2.9·10^-01^ | 1.0·10^-03^ | 7.2·10^-02^ | 1.0·10^-04^ | 6.4·10^-01^ |
| **WT ABA TR (7d)** | 2.3·10^-08^ | 1.8·10^-06^ | N.A. | 9.7·10^-07^ | 1.6·10^-09^ | 3.5·10^-08^ | 4.9·10^-02^ | 2.9·10^-05^ |
| **WT PAC (7d)** | 5.0·10^-01^ | 4.6·10^-02^ | 1.9·10^-06^ | N.A. | 1.3·10^-01^ | 4.5·10^-01^ | 1.4·10^-04^ | 2.5·10^-01^ |
| ***abi3-8* 3 HAI** | 2.9·10^-02^ | 1.7·10^-03^ | 2.8·10^-08^ | 5.5·10^-02^ | N.A. | 1.6·10^-02^ | 1.9·10^-06^ | 2.3·10^-02^ |
| ***abi3-8* TR** | 9.9·10^-01^ | 7.2·10^-02^ | 3.5·10^-08^ | 6.4·10^-01^ | 1.6·10^-02^ | N.A. | 3.0·10^-05^ | 3.7·10^-01^ |
| **abi3-8 ABA TR (7 d)** | 1.4·10^-05^ | 1.0·10^-04^ | 1.3·10^-01^ | 7.4·10^-05^ | 1.9·10^-06^ | 3.0·10^-05^ | N.A. | 7.4·10^-04^ |
| **abi3-8 PAC TR (7 d)** | 3.8·10^-01^ | 6.4·10^-01^ | 8.7·10^-05^ | 6.9·10^-01^ | 2.3·10^-02^ | 3.7·10^-01^ | 5.2·10^-04^ | N.A. |

**Supplementary Table 3.** P-values for pairwise comparisons of radicle lengths as shown in Figure 4B & C. A two-tailed Student’s t-test was performed for each pair, and the respective p-value is given.

|  | **WT 3 HAI** | **WT PAC (7 d)** | **WT ABA TR (7 d)** | ***krp6-1* 3 HAI** | ***krp6-1* PAC TR (7 d)** | ***krp6-1* ABA TR (7 d)** |
| --- | --- | --- | --- | --- | --- | --- |
| **WT 3 HAI** | N.A. | 2.4·10^-01^ | 6.4·10^-01^ | 8.0·10^-01^ | 1.1·10^-02^ | 1.4·10^-03^ |
| **WT PAC (7 d)** | 2.4·10^-01^ | N.A. | 2.5·10^-06^ | 3.7·10^-01^ | 6.0·10^-02^ | 7.0·10^-03^ |
| **WT ABA TR (7 d)** | 6.4·10^-07^ | 2.5·10^-06^ | N.A. | 1.6·10^-07^ | 1.4·10^-04^ | 4.5·10^-03^ |
| ***krp6-1* 3 HAI** | 8.0·10^-01^ | 3.7·10^-01^ | 1.6·10^-07^ | N.A. | 1.1·10^-02^ | 9.5·10^-04^ |
| ***krp6-1* PAC TR (7 d)** | 1.1·10^-02^ | 6.0·10^-02^ | 1.4·10^-04^ | 1.1·10^-02^ | N.A. | 2.2·10^-01^ |
| ***krp6-1* ABA TR (7 d)** | 2.5·10^-04^ | 7.0·10^-03^ | 4.5·10^-03^ | 1.6·10^-04^ | 2.2·10^-01^ | N.A. |
